# Survival of the fattest

**DOI:** 10.7554/eLife.01760

**Published:** 2013-11-26

**Authors:** Sophie M Morgani, Joshua M Brickman

**Affiliations:** 1 **Sophie M Morgani** is in the Danish Stem Cell Center (DanStem), University of Copenhagen, Copenhagen, Denmarksophie.morgani@sund.ku.dk; 2 **Joshua M. Brickman** is in the Danish Stem Cell Center (DanStem), University of Copenhagen, Copenhagen, Denmarkjoshua.brickman@sund.ku.dk

**Keywords:** Cell fate choice, Lineage priming, Salt and pepper, differentiation, heterogeneous, RAS, Dictyostelium

## Abstract

Experiments on the social amoeba *Dictyostelium discoideum* show that the origins of lineage bias in this system lie in the nutritional history of individual cells. Clues to the molecular basis for this process suggest similar forces may be at work in early mammalian development.

**Related research article** Chattwood A, Nagayama K, Bolourani P, Harkin L, Kamjoo M, Weeks G, Thompson CRL. 2013. Developmental lineage priming in *Dictyostelium* by heterogeneous Ras activation. *eLife* **2**:e01067. doi: 10.7554/eLife.01067

**Image** Wild-type (left) and mutant Dictyostelium discoideum showing the spore (top) and stalk

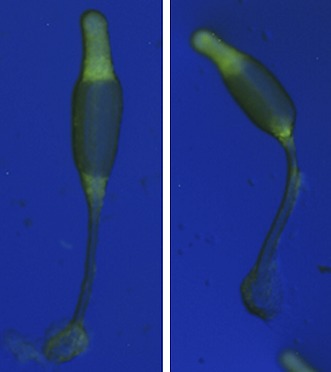

One of the fundamental questions in developmental biology is how a pattern can be generated within a group of seemingly identical cells. In many animals, including non-mammalian vertebrates, pattern is determined by the position of specific factors inside the mother’s egg. However, the initiation of patterns in many other species, including mammals and social amoeba, is not understood. When cells of *Dictyostelium discoideum*, a widely studied amoeba, are deprived of nutrients, they aggregate and differentiate to form a multicellular fruiting body comprised of a stalk and a number of spores. As in the early mammal, differences between the cells emerge seemingly at random. However, these initial differences are not fixed, and although the cells exhibit a bias towards a particular fate, a phenomenon known as ‘lineage priming’, they still retain the ability to become stalk or spore cells.

Lineage priming could arise within a population of cells as a result of stochastic bursts of gene expression (Elowitz et al., 2002) or through a deterministic element such as a molecular oscillator, the cell cycle or physical location. Feedback pathways, both negative and positive, could then amplify these early variations. However, there is another possibility that lies somewhere between these two alternatives. In *Dictyostelium*, the apparently ‘random’ fate of individual cells is influenced by their nutritional history. Cells grown without glucose are more likely to become stalk cells because they are more responsive to DIF, an extracellular signalling molecule that encourages the development of stalk cells, whereas cells grown with glucose tend to become spore cells (Leach et al., 1973; Thompson and Kay, 2000). This bias is only observed when cells with different nutrient histories are mixed together.

Now in eLife, Christopher Thompson and colleagues at Manchester University and the University of British Columbia—including Alex Chattwood and Koki Nagayama as joint first authors—provide a mechanism linking the nutritional history of cells to lineage priming (Chattwood et al., 2013). They identified the protein GefE, a guanine nucleotide exchange factor, as being critical in regulating this process. When mutant cells that contain very little GefE were allowed to develop on their own, they still showed a normal bias based on their exposure to glucose. However, when these same mutant cells were grown without glucose and mixed with normal cells grown in glucose, they were no longer biased towards a stalk cell fate.

The explanation for this competitive loss of lineage bias was that GefE mutants were shown to be less likely to respond to the DIF signalling molecule. Chattwood, Nagayama and colleagues found that GefE activates a protein called RasD that in turn dictates responsiveness to DIF. They showed that RasD expression differed between cells, changed over time, and was increased by a lack of glucose. When cells with different levels of RasD were mixed, those with low levels of RasD mostly became spore cells, whereas cells with high RasD levels exhibited a preference for becoming stalk cells.

The role of DIF in *Dictyostelium*, promoting one cell fate over another, is comparable to the role of the growth factor FGF in mice. In early mouse development some cells will become primitive endoderm (PrE) cells—cells that support the embryo—and others become epiblast (i.e., they become part of the embryo). FGF encourages cells to become PrE (Yamanaka et al., 2010). However, before the cells segregate into these distinct lineages, precursor cells already exist that exhibit bias when challenged to differentiate in competition with other cells (Canham et al., 2010; Grabarek et al., 2012). PrE precursors have been shown to express higher levels of the FGFR2, the receptor for factor FGF, which renders them more likely to respond to this growth factor.

In *Dictyostelium*, DIF activates the evolutionarily conserved JAK/STAT pathway, while in mice the homolog of RasD is a component of the Fgf pathway, positioned just downstream of the FGF receptor. Interestingly, both of these pathways are involved in the formation of extraembryonic lineages in early mouse development (Stewart et al., 1992; Yamanaka et al., 2010) and embryonic stem cells (Morgani et al., 2013). Is it a coincidence that species as distantly related as *Dictyostelium* and mammals use the same pathways to modulate probabilistic cell fate choice? It is also noteworthy that the stalk cells in amoeba and PrE cells in mice both give rise to supportive structures that aid in the future development of the organism.

Perhaps the answer to this question lies in thinking about what advantages a historical lineage bias offers over either stochastic or traditional deterministic models. In the case of *Dictyostelium*, bequeathing the most nutritionally healthy cells to the next generation through the spore increases survival odds. A model incorporating historical bias into cell fate choice (Figure 1) would ensure that spores could be formed in all cases but, when fitter cells were present, they would be favoured for spore generation. Does the conservation of signalling pathways suggest a similar competitive mechanism is at work in the early mammalian embryo?

**Figure 1.**
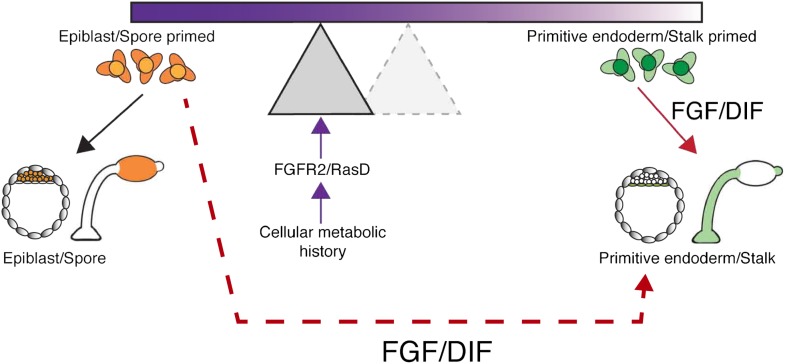
Potential mechanisms for introducing bias into lineage priming. When the amoeba *D. discoideum* is deprived of nutrients, it responds by forming a multicellular fruiting body made up of stalk and spore cells. The decision to become a stalk cell is induced by the signalling molecule DIF, and the nutrient history of each cell influences the likelihood that it will respond to DIF and adopt a particular fate. A similar situation arises in the early mouse embryo where cells can choose between becoming epiblast or primitive endoderm (PrE) cells, with the growth factor FGF promoting a PrE fate. The process can be visualized as a seesaw, with the nutrient history influencing the position of the fulcrum and, therefore, the likelihood that a cell will tipped towards a particular fate when exposed to DIF or FGF. In this way, the nutrient history of individual cells can establish precursor populations that are likely to become a stalk cell or a spore cell. Chattwood, Nagayama and co-workers now show that nutrient history affects the level of a protein called RasD in *Dictyostelium*, which in turn influences the ability of a cell to respond to DIF: high levels of RasD correspond to the fulcrum being closer to the left end of the seesaw (shaded triangle), so that the cells are primed to become stalk cells and exposure to DIF (solid red arrow) is more likely to lead to them becoming stalk cells. However, the primed state of these cells is not fixed because RasD expression can change based on nutrient history and the fulcrum can return to the right (dashed triangle), where cells are primed to become spore cells. When the fulcrum is positioned on the right, the cells are primed, but their fate is not determined because they can still become stalk cells if treated with high levels of DIF (dashed red arrow). In mice, a receptor called FGFR2 and a signalling molecule called FGF influence cell fate in a similar way to RasD and DIF in Dictyostelium. This suggests the intriguing possibility that nutrient history could influence the choice between epiblast and PrE lineages.

In essence, lineage priming is an experimental measurement of the ability of cells to compete for a particular lineage choice. While there is no evidence of direct competition for a particular lineage, recent work suggests that epiblast cells can sense their defective neighbours and outcompete them (Sancho et al., 2013). In this case, cells sense a genetically defective neighbour but thus far there is no evidence that mammalian cells can judge the metabolic fitness of a genetically normal neighbour. However, the first clear example of cell competition, observed in *Drosophila*, was based upon the relative metabolic activity of cells (Morata and Ripoll, 1975).

Intriguingly, the first lineage decisions, in the earliest stages of mouse embryonic development, coincide with a switch to glucose metabolism (Wales et al., 1995). Perhaps probabilistic lineage bias ensures that the metabolically fittest cells adopt an embryonic fate. How then does RasD impact on the probability of cells to respond to DIF? This is an important, unanswered question that could shed light on the evolution of competitive mechanisms to register metabolic fitness through signalling pathways that might also be conserved in early mammalian development.
